# The Contribution of Nitrate Dissimilation to Nitrate Consumption in *narG*- and *napA*-Containing Nitrate Reducers with Various Oxygen and Nitrate Supplies

**DOI:** 10.1128/spectrum.00695-22

**Published:** 2022-12-01

**Authors:** Xing Chen, Chunmei Liu, Baoli Zhu, Wenxue Wei, Rong Sheng

**Affiliations:** a Key Laboratory of Agro-ecological Processes in Subtropical Regions and Taoyuan Agro-ecosystem Research Station, Soil Molecular Ecology Section, Institute of Subtropical Agriculture, Chinese Academy of Sciences, Changsha, China; b University of Chinese Academy of Sciences, Beijing, China; Huazhong Agricultural University

**Keywords:** nitrate reducers, *narG*, *napA*, oxygen, nitrate, nitrate reducer

## Abstract

Nitrate reducers containing *narG* or *napA* play an important role in the nitrogen cycle, but little is known about their functional differentiations in relation to environmental changes. In this study, three types of nitrate reducers in the genus Pseudomonas, including strains containing *narG* (G type), *napA* (A type) and both *narG* and *napA* (GA type), were selected to explore their functional performances under varied nitrate and oxygen concentrations. Their growth characteristics, nitrate consumption, and dissimilatory nitrate-reducing activity were investigated. Growth and nitrate consumption of all three types of strains were generally promoted with increasing oxygen and nitrate concentrations. However, their dissimilatory nitrate-reducing activities were restricted by oxygen supply. When supplied with 0.25 mM KNO_3_, A-type strains showed a higher growth rate but lower activity of dissimilatory nitrate reduction (DNR) than G-type strains, regardless of oxygen concentration. However, when nitrate concentration increased to 0.75 mM or 5 mM, G-type strains displayed stronger capability of nitrate consumption and DNR than A-type strains under anaerobic conditions, whereas under oxygenated conditions, A-type strains exhibited higher growth and nitrate consumption but weaker DNR than G-type strains. The GA-type strains appeared similar to G type under anaerobic conditions but performed more similarly to A type in aerobic environments. In summary, the nitrate consumption of *narG*-containing nitrate reducers is mainly caused by DNR in both anaerobic and aerobic environments, while the large proportion of nitrate consumption in A-type nitrate reducers under the aerobic condition is attributed to the assimilation by cell growth.

**IMPORTANCE** Nitrate reducers containing *narG* or *napA* are ubiquitous, but little is known about their functional performance in various environments. Our study provides an important clue that the nitrate consumption of *narG*-containing strains is mainly caused by dissimilatory reduction in the environments, while that of *napA*-containing nitrate reducers under anaerobic conditions is ascribed to nitrate dissimilation but under the aerobic condition is attributed to the assimilation by cell growth. This finding broadens the understanding of aerobic nitrate reduction in the nitrogen cycle and highlights the important role of *narG*-containing bacteria in nitrate reduction under aerobic conditions.

## INTRODUCTION

The dissimilatory reduction of nitrate to nitrite by bacteria, the first step of denitrification and dissimilatory nitrate reduction to ammonium (DNRA), is an important process in nitrogen cycling. The nitrate respiration may increase the risk of nitrogen loss from agricultural ecosystems, as well as the emission of greenhouse gas (1–3). Dissimilatory nitrate reduction is catalyzed by two types of nitrate reductase, membrane-bound nitrate reductase (Nar) and periplasmic nitrate reductase (Nap). The active subunits of these two isozymes are encoded by the *narG* and *napA* genes, respectively (4–6). Most nitrate-reducing bacteria contain only *narG* or *napA*, but some strains contain both (7, 8).

Nitrate reducers with *narG* are widely distributed in diverse bacterial phyla, including *Proteobacteria*, *Firmicutes*, and *Bacillus*, and also *Archaea* (9–11), but the nitrate reducers with *napA* mainly belong to *Proteobacteria* (12). Moreover, Nar is composed of a cytoplasmic (NarGH) complex and a membrane anchor subunit (NarI), while Nap is a soluble protein, mainly consisting of NapA and NapB subunits (4, 5). These differential characteristics of the two types of nitrate reductase imply that their contributions to nitrate reduction may also vary across ecosystems or environmental conditions. Understanding the functional differences of bacteria with different nitrate reductases will provide insight into their potential ecological significance for predicting nitrate reduction in the environment.

The abundances and community compositions of bacteria containing *narG* or *napA* have been extensively studied in a variety of ecosystems (9, 10, 13, 14). It was reported that the abundance of *narG* in river or flooded paddy soil samples was much higher than that of *napA* (15, 16), implying that *narG*-containing bacteria may prefer mionectic environments. In contrast, the relative abundance of *napA* tended to increase with increasing dissolved oxygen in the biofilm (17, 18). In a loose soil environment with good air permeability, the copy number of the *napA* gene was even higher than that of the *narG* gene (19, 20). Several studies of nitrate-reducing strains also reported that Nar was associated with anaerobic nitrate respiration under high-nitrate conditions (21, 22), while Nap was considered to play an important role in aerobic nitrate reduction, especially at low nitrate concentrations (23, 24). It seems that *narG*- and *napA*-containing bacteria may have distinct niches, and the variations in oxygen and nitrate concentration may contribute to the niche differentiation between *narG*- and *napA*- containing nitrate reducers. However, some studies suggested that Nar was also involved in aerobic nitrate reduction (8) and Nap could also perform nitrate reduction under anaerobic conditions (25–27). Thus, it is unclear how the functional activities of nitrate reducers containing *narG*, *napA*, or both *narG* and *napA* respond to oxygen and nitrate supply.

In this study, three types of Pseudomonas isolates, i.e., strains containing *narG* (G type), *napA* (A type), or both *narG* and *napA* (GA type), were selected. The aim of the investigation was to assess the functional differentiations of the three types of nitrate reducers under varied oxygen and nitrate concentrations.

## RESULTS

### Dynamics of cell growth.

**FIG 1:**
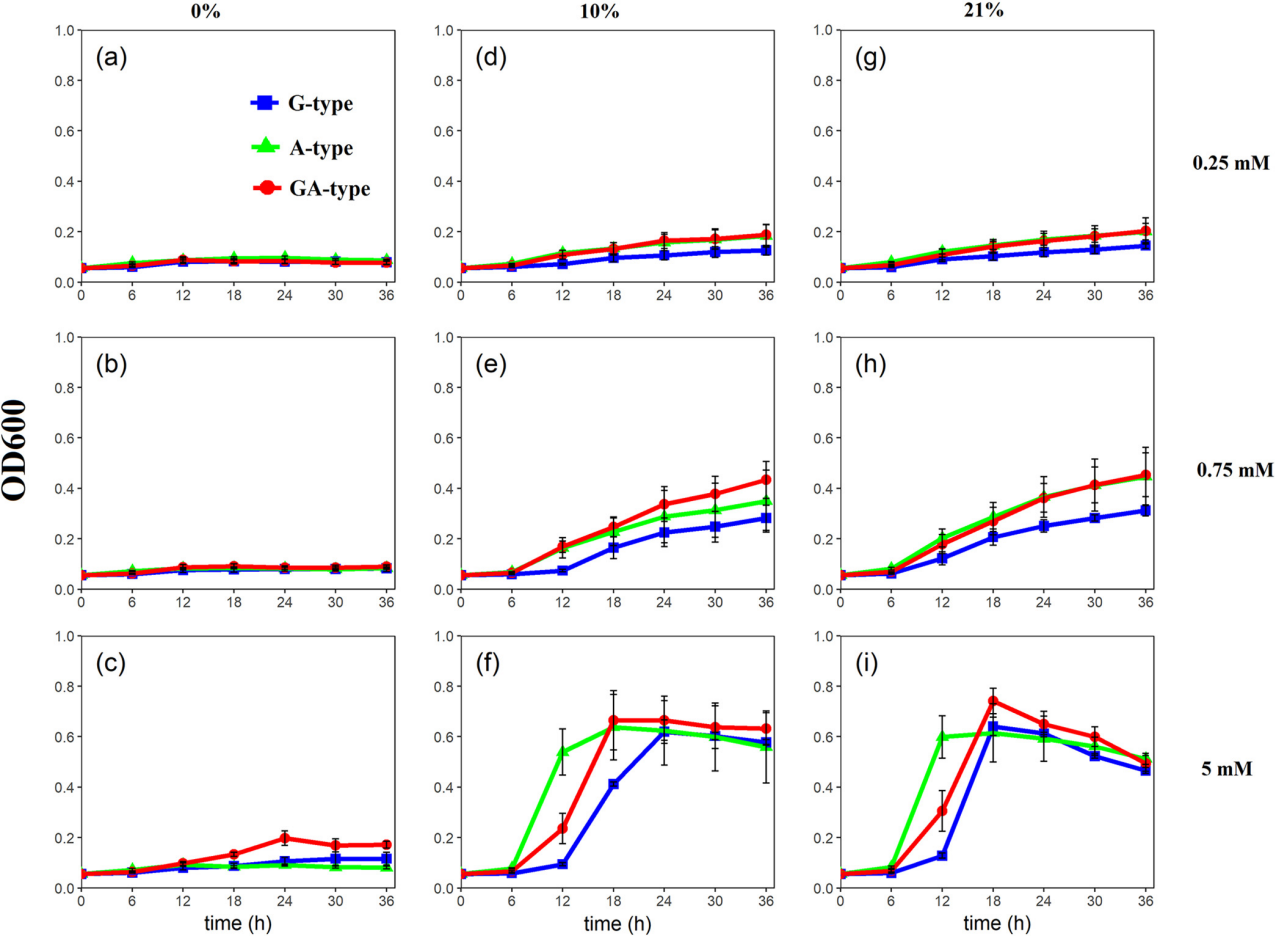
Cell growth of three types of nitrate reducers under varied oxygen and nitrate concentrations. Strains were cultured under anaerobic conditions (0% O_2_) with 0.25 mM KNO_3_ (a), 0.75 mM KNO_3_ (b), and 5 mM KNO_3_ (c), under facultative conditions (10% O_2_) with 0.25 mM KNO_3_ (d), 0.75 mM KNO_3_ (e), and 5 mM KNO_3_ (f), and under aerobic conditions (21% O_2_) with 0.25 mM KNO_3_ (g), 0.75 mM KNO_3_ (h), and 5 mM KNO_3_ (i). Standard errors for each type of nitrate reducer (*n* = 9, 3 strains × 3 replicates) are given.

It was shown that the growth rates of all the strains were regulated by nitrate concentration under all redox conditions tested (Fig. 1; Table 1). Generally, the higher NO_3_^−^ concentration supported the higher growth rate. Under anaerobic conditions (0% O_2_), all the strains of G, A, and GA type grew very slowly (Fig. 1a, b, and c). The growth rates among the three types were similar without significant differences under three nitrate inputs, except that the GA type displayed a significantly (*P* < 0.05) higher growth trend than those of G type and A type under 5 mM NO_3_^−^ (Fig. 1c). However, when O_2_ was increased either to 10% (Fig. 1d, e, and f) or to 21% (Fig. 1g, h, and i), the growth of all strains was significantly promoted, regardless of nitrate concentration. Interestingly, the dynamics of the growth of all strains in 10% O_2_ treatments were quite similar to those in 21% O_2_ treatments, which were characterized by the growth rates of G type, on average, always being the lowest during the incubation. GA-type strains exhibited similar growth trends as A type under 0.25 mM and 0.75 mM NO_3_^−^, but under 5 mM NO_3_^−^, the strains of A type grew significantly (*P* < 0.05) faster than GA type during 0 to 12 h.

**TABLE 1:** Average growth rate of three types of strains under varied nitrate and oxygen concentrations

Oxygen concn (%, vol/vol)	Nitrate concn (mM)	Avg growth rate (OD units h^−1^) for strain type^*a*^:		
		G type	A type	GA type
0	0.25	0.0014a	0.0020a	0.0020a
	0.75	0.0021a	0.0027a	0.0028a
	5	0.0021b	0.0030a	0.0035a

10	0.25	0.0015b	0.0050a	0.0044a
	0.75	0.0020b	0.0093a	0.0094a
	5	0.0030c	0.0403a	0.0144b

21	0.25	0.0029b	0.0054a	0.0047a
	0.75	0.0055b	0.0123a	0.0103ab
	5	0.0065c	0.0453a	0.0208b

a The different letters indicate significant differences (*P *< 0.05) between three types of nitrate-reducing strains with certain oxygen and nitrate supplies according to Duncan’s test. The growth rate was calculated based on the slope in 0 to 12 h.

### Dynamics of nitrate consumption.

**FIG 2:**
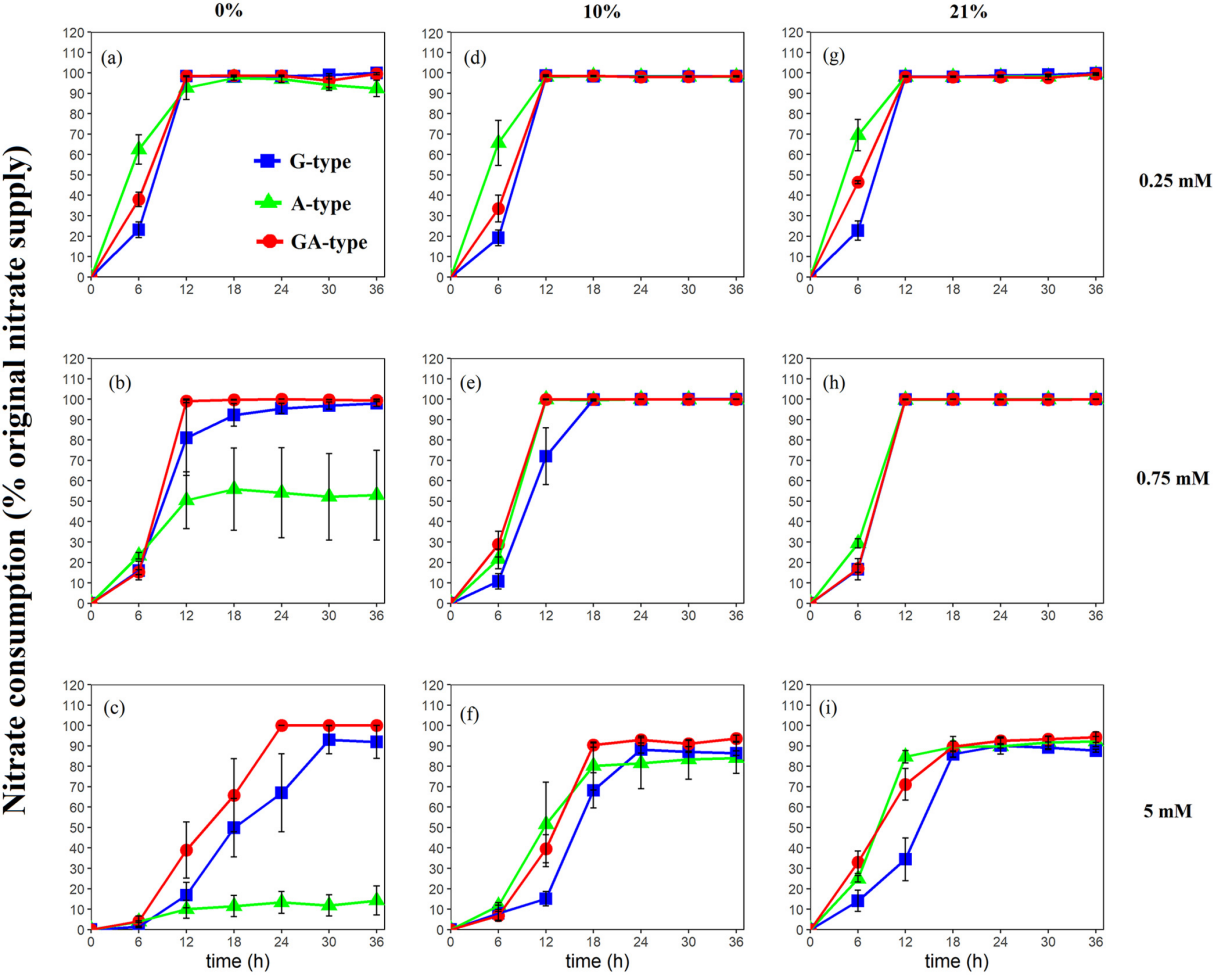
Nitrate consumption of three types of nitrate reducers during incubation. Strains were cultured under anaerobic conditions (0% O_2_) with 0.25 mM KNO_3_ (a), 0.75 mM KNO_3_ (b), and 5 mM KNO_3_ (c), under facultative conditions (10% O_2_) with 0.25 mM KNO_3_ (d), 0.75 mM KNO_3_ (e), and 5 mM KNO_3_ (f), and under aerobic conditions (21% O_2_) with 0.25 mM KNO_3_ (g), 0.75 mM KNO_3_ (h), and 5 mM KNO_3_ (i). Standard errors for each type of nitrate reducer (*n* = 9, 3 strains × 3 replicates) are given.

The nitrate consumption rates (NCRs) of the strains during the incubation were clearly affected by redox status (Fig. 2 and Table 2; see also Fig. S2 in the supplemental material). When the microorganisms were grown in an anaerobic environment, the nitrate supply led to dynamic variations in the NCRs by the three types of strains. With 0.25 mM NO_3_^−^, there was no significant difference in NCRs among the three types of strains at 12 h (Table 2), but the NCR of A type was significantly higher than that of G type at 6 h. However, when NO_3_^−^ input was increased to 0.75 mM, the NCRs of G-type strains were significantly higher than those of A type after 12 h of incubation, which the maximum nitrate consumption of G- and A-type strains reached 100% and 55% of nitrate supply, respectively, and the nitrate consumption of GA type was even slightly higher than that of G type. When the initial NO_3_^−^ concentration further increased to 5 mM, it was interestingly found that the A-type strains consumed only 10% of the nitrate supply, but G type consumed 90%, and the NCRs of GA type were significantly higher than those of G type. However, when the growth medium was oxygenated with 10% and 21% O_2_, A-type strains possessed significantly higher NCRs than G type in 5 mM NO_3_^−^ (Table 2). In comparison with anaerobic conditions, the nitrate consumption of A-type strains dramatically increased to 90% to 100% of nitrate supply when they were supplied with 0.75 mM and 5 mM NO_3_^−^. In contrast, G-type strains presented a similar trend as that in anaerobic treatments. The NCRs of GA-type strains in aerobic environments showed no significant difference from A and G types at 0.25 mM NO_3_^−^ but presented values closer to A type at 0.75 mM and 5 mM NO_3_^−^.

**TABLE 2:** Average nitrate consumption rate of three types of strains under varied nitrate and oxygen concentrations

Oxygen concn (%, vol/vol)	Nitrate concn (mM)	Avg nitrate consumption rate (mg N L^−1^ h^−1^) for strain type^*a*^:		
		G type	A type	GA type
0	0.25	0.27a	0.24a	0.27a
	0.75	0.63a	0.39b	0.78a
	5	0.94b	0.56b	2.18a

10	0.25	0.27a	0.26a	0.27a
	0.75	0.58b	0.80a	0.80a
	5	1.00b	2.89a	2.20ab

21	0.25	0.27a	0.26a	0.27a
	0.75	0.78b	0.78b	0.81a
	5	2.20c	4.83a	3.96b

a The different letters indicate significant differences (*P *< 0.05) between three types of nitrate-reducing strains with certain oxygen and nitrate supplies according to Duncan’s test. The nitrate consumption rate was calculated based on the slope in 0 to 12 h.

### Dissimilatory nitrate-reducing activity.

**FIG 3:**
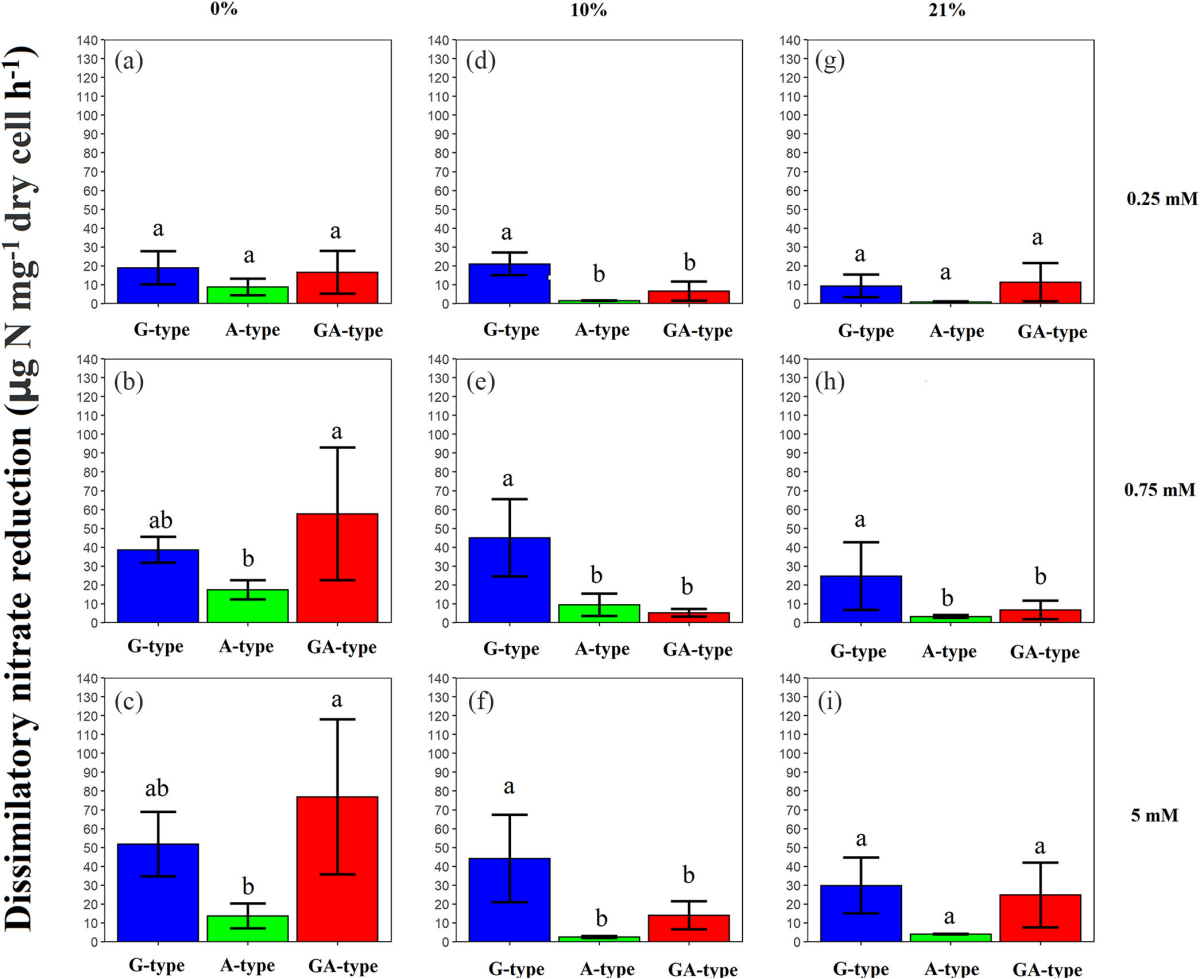
The dissimilatory nitrate-reducing activity of three types of nitrate reducers under varied nitrate and oxygen concentrations. Strains were cultured under anaerobic conditions (0% O_2_) with 0.25 mM KNO_3_ (a), 0.75 mM KNO_3_ (b), and 5 mM KNO_3_ (c), under facultative conditions (10% O_2_) with 0.25 mM KNO_3_ (d), 0.75 mM KNO_3_ (e), and 5 mM KNO_3_ (f), and under aerobic conditions (21% O_2_) with 0.25 mM KNO_3_ (g), 0.75 mM KNO_3_ (h), and 5 mM KNO_3_ (i). Standard errors for each type of nitrate reducer (*n* = 9, 3 strains × 3 replicates) are given. The data between 0 and 12 h were used to estimate the activities of dissimilatory nitrate reduction.

It was found that G-type strains generally displayed higher dissimilatory nitrate-reducing activities than A-type strains under varied oxygen and nitrate concentrations, while GA-type strains displayed slightly higher activity than G type under anaerobic conditions but significantly lower activity under aerobic conditions. In anaerobic environments (Fig. 3a, b, and c), the increase of nitrate input obviously enhanced the dissimilatory nitrate-reducing activities of all strains, but the G- and GA-type strains responded more significantly than A type. For instance, the dissimilatory nitrate-reducing ability of G type with 5 mM NO_3_^−^ was 2.7 times that under 0.25 mM NO_3_^−^, while it was 1.6 times for A type. Although GA type possessed the highest nitrate-reducing ability under 5 mM NO_3_^−^ (Fig. 3c), there were no significant differences between G type and GA type.

When the strains were exposed to 10% O_2_, some strains of the G type maintained high nitrate-reducing activities like those under anaerobic conditions (Table S1), but the DNR activities of A-type strains were significantly reduced to 1.5 to 9.5 μg N mg^−1 ^dry cell h^−1^ (reduced by 63% to 83%, compared with 0% O_2_). However, when the O_2_ concentration further increased to 21% (Fig. 3g, h, and i), although the nitrate-reducing activities of G-type strains were also decreased (reduced by 41% to 51%, compared to anaerobic condition), they still possessed the activity of 9.3 to 29.8 μg N mg^−1 ^dry cell h^−1^, while those of A-type strains were further reduced to 0.8 to 4.0 μg N mg^−1 ^dry cell h^−1^. Therefore, in an aerobic environment, G-type strains still maintained a certain level of dissimilatory nitrate-reducing ability while this function of A-type strains was negligible. It was also detected that the functional performances of GA-type strains under oxygenated conditions with various nitrate supplies were between those of G and A types.

### The relative contribution of dissimilatory nitrate reduction.

**FIG 4:**
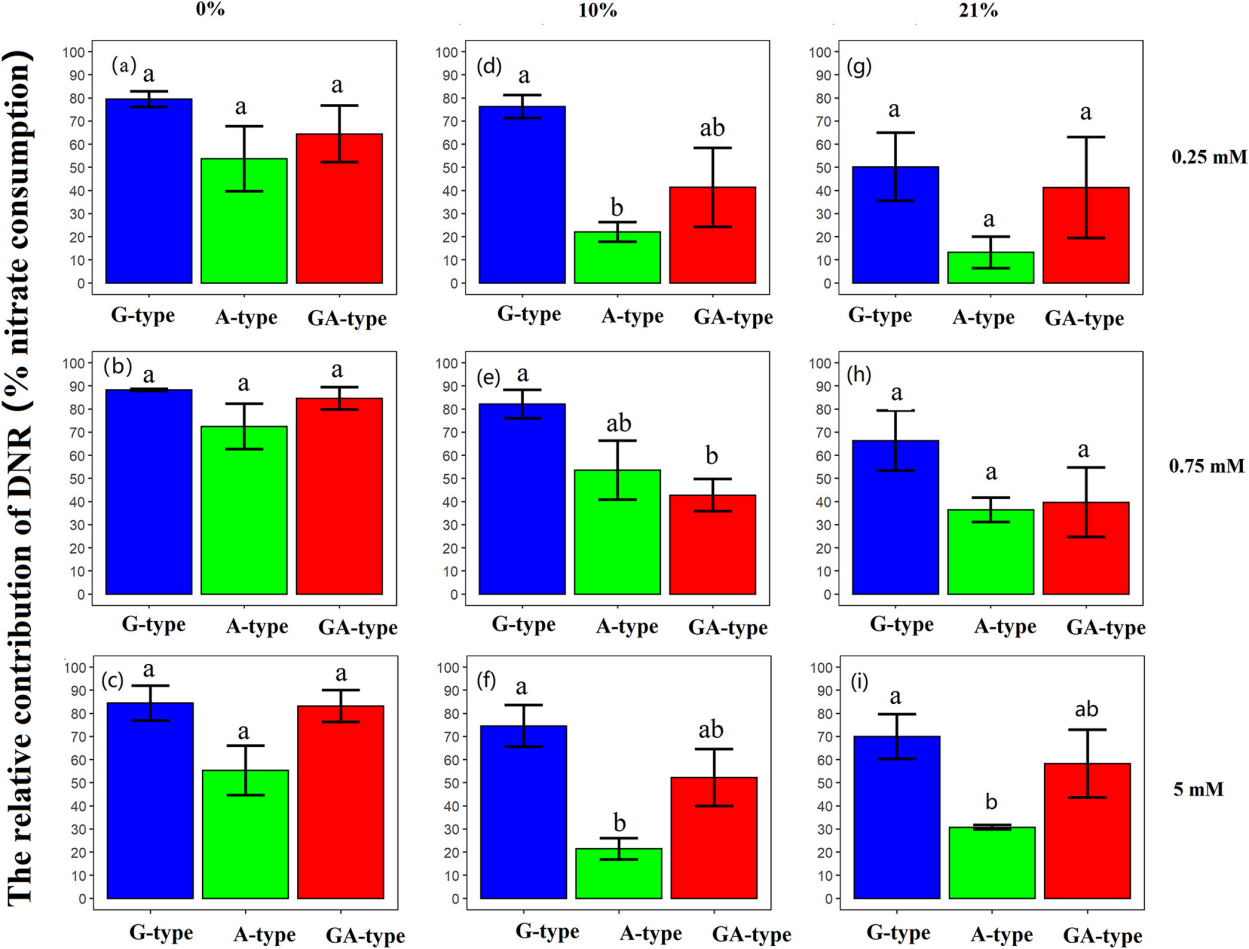
The relative contribution of dissimilatory nitrate reduction under varied nitrate and oxygen concentrations. Strains were cultured under anaerobic conditions (0% O_2_) with 0.25 mM KNO_3_ (a), 0.75 mM KNO_3_ (b), and 5 mM KNO_3_ (c), under facultative conditions (10% O_2_) with 0.25 mM KNO_3_ (d), 0.75 mM KNO_3_ (e), and 5 mM KNO_3_ (f), and under aerobic conditions (21% O_2_) with 0.25 mM KNO_3_ (g), 0.75 mM KNO_3_ (h), and 5 mM KNO_3_ (i). DNR indicates the dissimilatory nitrate reduction. Standard errors are indicated (*n* = 9, 3 strains × 3 replicates) for each type of nitrate reducer. Different letters indicate significant differences (*P* < 0.05) among types. The data between 0 and 12 h were used to determine the contribution of dissimilatory nitrate reduction.

The results showed that the proportions of dissimilatory nitrate reduction (DNR) of G, A, and GA types clearly differed among different culture conditions (Fig. 4). It was found that the contribution of DNR in G-type strains was dominant in nitrate consumption under all conditions, ranging from 50% to 88%. The second place was taken by GA type, with proportions between 40% and 80%, while A type possessed the lowest proportions. The highest proportion of DNR for each type strain coincidentally occurred under anaerobic conditions, regardless of the nitrate concentration. The G-, A-, and GA-type strains similarly maintained their proportions with 80 to 88%, 54 to 70%, and 65 to 84%, respectively (Fig. 4a, b, and c). When the growth media were oxygenated with 10% and 21% O_2_, the proportions of DNR in G-type strains dropped by an average of 6.4% and 21.9% compared to that in anaerobic treatments, respectively. The A-type strains responded more strongly to the oxygenation than did G type; their proportions of DNR sharply decreased by about 28% in 10% O_2_ treatments and 34% in 21% O_2_ treatments. The strains of GA type performed slightly differently from those of G and A types: they were characterized by a sudden reduction of DNR contribution with 32% when facing 10% O_2_, but the further rise of O_2_ concentration did not induce clear shifts under different nitrate concentrations.

## DISCUSSION

Nar and Nap are two isozymes that are responsible for dissimilatory nitrate reduction and coexist in natural ecosystems. Their active subunits are encoded by *narG* and *napA* genes, respectively, but little is known about their functional performance in various environments. In the present study, it was observed that the contributions of dissimilatory nitrate reduction for nitrate consumption accounted for 60% to 84% on average in different types of nitrate-reducing strains under anaerobic conditions (Fig. 4a, b, and c), suggesting that the nitrate consumption under anaerobic environments would be mainly attributed to dissimilatory reduction. In accord with our results, previous studies also documented that the dissimilatory nitrate reduction is more likely to occur in anaerobic environments (23, 28). However, nitrate respiration is a lower-energy-yielding respiration system than oxygen respiration (29–31), resulting in lower growth yield under anaerobic conditions (Fig. 1a, b, and c). In addition, the isolation of strains under aerobic conditions may be another important reason for their poor growth under anaerobic conditions. More interestingly, A-type bacteria had the lowest activity of DNR under anaerobic conditions with varied nitrate concentration (Fig. 3), which may be related to the fact that they contain only the dissimilatory nitrate reductase *napA* gene (see Table S2 in the supplemental material). Besides, the dissimilatory nitrate-reducing activities of A-type strains were not significantly improved with the increasing nitrate concentration, while those of G-type strains maximally increased 2.7 times in 5 mM nitrate compared to 0.25 mM nitrate (Fig. 3a, b, and c). This phenomenon might be closely associated with the distinct regulatory systems for *nar* and *nap* gene expression (24, 32). The *nap* operon was reported to encode a “low-substrate-induced” reductase that is maximally expressed only at low levels of nitrate but would be suppressed under high-nitrate conditions. In contrast, the *nar* operon is maximally expressed when nitrate concentration is high but is weakly expressed at low nitrate levels (24, 26, 27). These results suggested that the G-type strains would play a more crucial role in nitrate reduction than A-type strains under anaerobic conditions, especially when the nitrate concentration was high.

In comparison, the presence of O_2_ could significantly improve bacterial growth (Fig. 1 and Table 1) but restricted the dissimilatory nitrate reduction (Fig. 3). This may explain why the abundance of nitrate reducers was higher in the top soil layer and decreased with depth but was weakly correlated with the nitrate-reducing activity (14). However, it is worth noting that A-type strains possessed higher growth rates and more biological nitrogen (bio-N) (Fig. 1 and Fig. S1) but a lower contribution of dissimilatory nitrate reduction for nitrate consumption than G type (Fig. 4) when they were exposed to O_2_. Therefore, we speculated that the greater nitrate consumption of A type under aerobic conditions could be largely attributed to the high nitrate assimilation caused by faster cell growth, while that of G-type strains still largely relied on dissimilatory reduction. From previous studies, it has been documented that Nap exhibits a higher substrate affinity than Nar (33, 34). In addition, Nap was also suggested not only to be responsible for anaerobic nitrate reduction but also to be conducive to cell growth and nitrate reduction under aerobic conditions (26, 27). These characteristics of Nap would facilitate the nitrate utilization and growth of A-type strains under aerobic conditions. This phenomenon is corroborated by a recent study by Li et al. (35), which showed that the large amount of nitrate removal by a Pseudomonas strain with *napA* in aerobic reactors was caused by microbial growth. However, the response pattern of G type seems to contradict previous reports that the expression of *narG* was mediated by the transcriptional regulator Fnr under anaerobic conditions (24, 36, 37). Besides Fnr, there are other protein regulatory systems involved in the process of dissimilatory nitrate reduction, including Anr, Dnr, and NarX-NarL (38, 39). Therefore, although we know little about the real mechanisms regarding the response of G-type strains toward oxygen, the high diversity of protein regulatory systems may help strains to maintain dissimilatory nitrate-reducing activity even under aerobic conditions. Actually, it has been documented that aerobic nitrate reduction can occur in diverse bacteria with multiple types of physiological controls (8), but further study should be conducted to explore the associated mechanism.

Furthermore, we observed that the nitrate-reducing activities of GA-type strains were comparable to those of G type rather than A type under anaerobic conditions, implying that the nitrate-reducing function of GA-type strains might mainly rely on the activity of Nar rather than Nap in anaerobic environments. A recent study conducted under anaerobic conditions also found that Pseudomonas strains with both *narG* and *napA* could not consume nitrate when the *narG* gene was knocked out (40). However, when they were exposed to oxygen, the nitrate consumption patterns of GA-type strains were generally between those of A- and G-type strains under varied nitrate concentrations, and GA-type strains acted more similarly to A-type strains. The mechanisms involved in regulating the functions of GA-type strains under aerobic conditions are still elusive and need further study. Although the selected strains could not represent all three types of nitrate reducers, the difference of nitrate-reducing capability among three types could provide evidence in the functional differentiation of nitrate-reducing bacteria under various environmental conditions. Also, further studies are required to explore the underlying mechanisms of different physiological performance between the partial and complete denitrifiers.

### Conclusion.

In conclusion, our study suggested that the nitrate consumption of all three types of nitrate reducers under anaerobic conditions was mainly caused by dissimilatory reduction. The presence of O_2_ decreased the contribution of dissimilatory reduction to nitrate consumption, but the nitrate consumption of G-type strains under aerobic conditions still largely relied on dissimilatory reduction while that of A type could be largely attributed to the nitrate assimilation caused by fast cell growth. Moreover, most G-type bacteria possessed higher activity of dissimilatory nitrate reduction than did A-type strains in both anaerobic and aerobic environments. The GA-type nitrate reducers acted similarly to G type under anaerobic condition but performed more similarly to A-type strains under aerobic conditions. Further investigations are required to reveal the real performance and associated mechanisms of different types of nitrate reducers in natural environments.

## MATERIALS AND METHODS

### Isolation of nitrate reducers.

The nitrate-reducing bacteria used in this study were isolated from a vegetable soil that had continuously received large amounts of fertilizers annually for over 10 years. The soil was derived from alluvial deposits and the field located at Changsha (113°01′ E, 28°26′ N), China. Soil samples (0 to 15 cm) were collected from multiple points in the field. After removal of visible debris and thorough mixing, the soil samples were transported immediately to the laboratory on ice. The enrichment medium (EM) used to isolate nitrate reducers consisted of the following components (per L): KNO_3_, 1.0 g; KH_2_PO_4_, 1.50 g; Na_2_HPO_4_·12H_2_O, 10.50 g; MgSO_4_·7H_2_O, 0.20 g; sodium succinate, 2 g; trace element solution, 1 mL. (The trace element solution contained the following in 100 mL: EDTA·2Na, 0.29 g; ZnSO_4_·7H_2_O, 1.095 g; MnSO_4_·H_2_O, 0.154 g; CuSO_4_·5H_2_O, 0.039 g; Na_2_MoO_4_·2H_2_O, 0.013 g; H_3_BO_3_, 0.012 g; FeSO_4_·7H_2_O, 0.5 g; CaCl·2H_2_O, 0.7 g.) Solid medium was made by adding 15 g agar per L.

The procedures for isolation under aerobic conditions referred to the work of Chen et al. (41). The detailed process was as follows. Fresh soil (20 g) was added to a 250-mL conical flask with 20 mL sterile water and then incubated for 10 days for starvation at 30°C and 120 rpm followed by 3 days without shaking. Afterward, 1 mL of the upper solution was transferred to a new sterile flask containing 30 mL fresh EM and incubated for 24 h at 30°C and 120 rpm; this process was repeated three times for enrichment. Finally, 100 μL of the suspension was inoculated on a plate with solid EM and cultured overnight at 30°C; then a single colony was transferred onto a solid EM plate with streaking successively three times to get pure strains. To avoid contamination, all the manipulations of inoculation and transfer were completed in a clean bench. All the medium and materials used in this experiment were sterilized by autoclaving for 20 min at 121°C.

### Identification of nitrate reducers.

First, the Griess reagent was used to primarily screen nitrate-reducing strains by determining nitrite production of the isolated strains (42). Subsequently, these primarily selected strains were cultured overnight in EM, and then the genomic DNA of each strain was extracted using a TIANamp bacterial DNA kit (TianGen Biotech, China). The dissimilatory nitrate reduction genes (*narG* and *napA*) and 16S rRNA of the isolates were amplified by PCR with the primers 145F/773R for *narG* (43), V16f/V17r for *napA* (44), and 8F/1492R for 16S rRNA (45, 46). The denitrification genes (*nirS*, *nirK*, *cnorB*, *qnorB*, and *nosZ*) were also determined. The PCR procedures were set up according to the work of Chen et al. (41). Then, PCR products were separated by electrophoresis on a 1.0% agarose gel and purified with a Tian gel midi-purification kit (TianGen Biotech, China). The amplified fragments were then sequenced by Sanger sequencing at Sangon Biotech (Shanghai, China), and the sequence homology analysis was conducted with BLASTN. The taxonomy analysis of isolated strains was performed based on the 16S rRNA gene sequencing.

**FIG 5:**
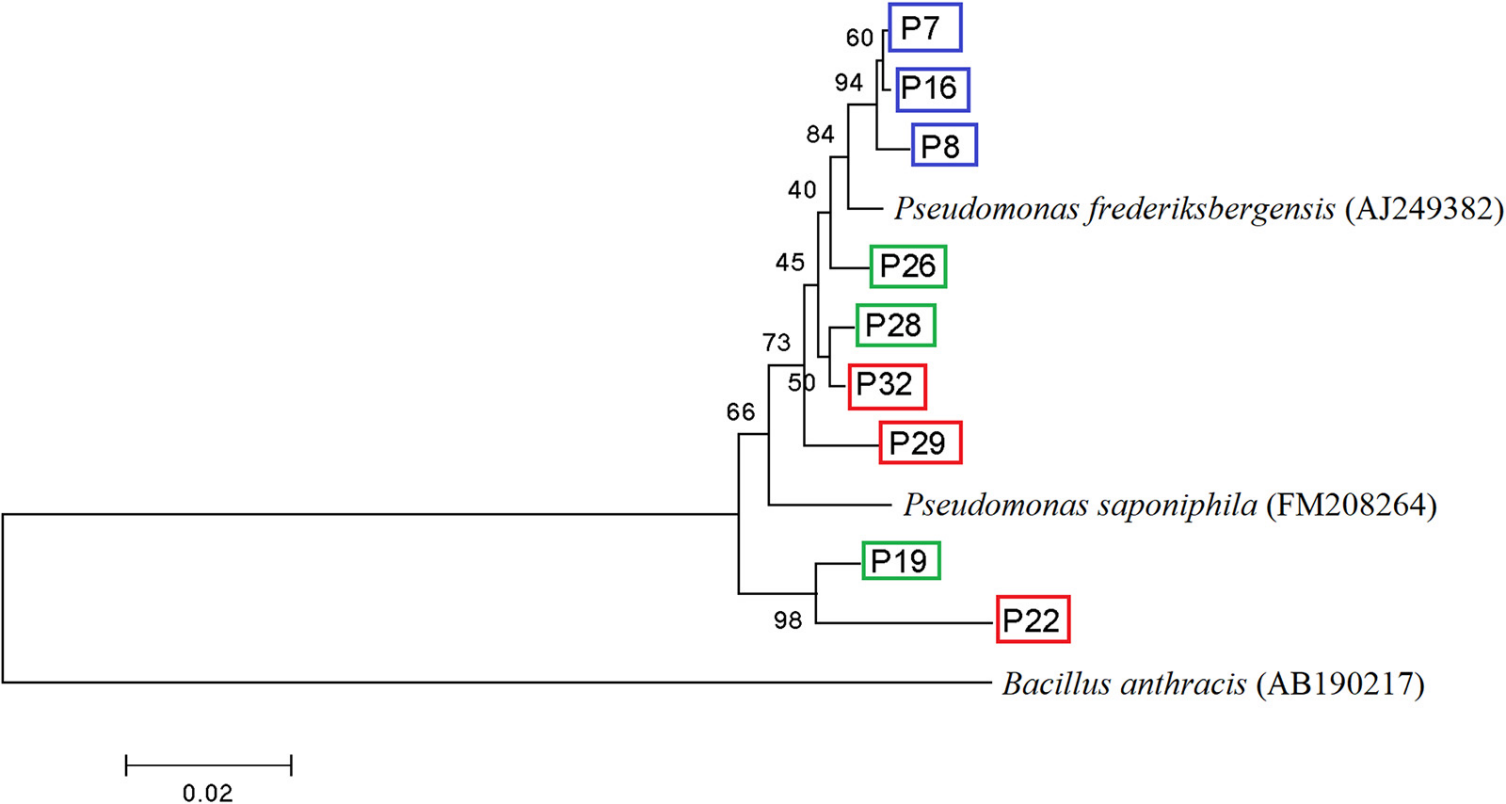
Phylogenetic relationships between selected strains. The phylogenetic tree is based on 16S rRNA gene sequences of strains, using the neighbor-joining method with MEGA 5.0 software based on 1,000 bootstrap replications. The sequence of Bacillus anthracis (AB190217) was used as the outgroup. The blue, green, and red boxes represent G-type, A-type, and GA-type isolates, respectively.

The strains that contained *narG*, *napA*, or both *narG* and *napA* genes were defined as G-type, A-type, and GA-type nitrate reducers, respectively. Since the nitrate reducers belonging to Pseudomonas are widely distributed in various ecosystems (7, 47), and three types of nitrate reducers were all available in this genus, a total of 9 Pseudomonas strains (3 strains × 3 types) were selected for further tests. The phylogenetic relationship of the 9 strains is shown in Fig. 5. The cooccurrence of other denitrification genes is presented in Table S2 in the supplemental material. P19, P26, and P28 had only the *napA* gene with no other denitrifying genes. P7, P8, and P16 had only the *narG* gene for a dissimilatory nitrate reductase gene, but P7 and P16 had full sets of denitrifying genes. P22, P29, and P32 had both *napA* and *narG* genes, with two strains (P29 and P32) having a complete set of denitrifying genes. The 16S rRNA and nitrate reductase gene sequences of the selected Pseudomonas strains are deposited in GenBank (see “Data availability” below).

### Cell preparation and culture experiment.

The isolates of G type (P7, P8, and P16), A type (P19, P26, and P28), and GA type (P22, P29, and P32) were preincubated in conical flasks containing 250 mL EM under aerobic conditions. When the cell growth reached logarithmic phase (about 12 h), the cell suspensions were centrifuged at 4,000 × *g* for 10 min. After two washes with 0.9% NaCl solution, the cell pellets were resuspended in 0.9% NaCl solution and then the cell densities were adjusted to 0.4 (optical density at 600 nm [OD_600_]). Subsequently, 1 mL of the adjusted suspension was transferred to three sealed cylinders (120 mL) which contained 100 mL functional characterization medium (FCM). The FCM contained the following (per L): trisodium citrate, 1.1 g; sodium succinate, 0.92 g; glucose, 0.68 g; KH_2_PO_4_, 1.50 g; Na_2_HPO_4_·12H_2_O, 10.50 g; MgSO_4_·7H_2_O, 0.20 g; trace element solution (as described above), 1 mL. Then, the inoculated FCM was divided into 21 cylinders (volume, 20 mL; diameter, 1.3 to 2.5 cm), with 5 mL medium per cylinder.

A total of nine treatments were set up with three nitrate levels and three oxygen concentrations for each strain. Three nitrate levels were achieved by adding KNO_3_ to FCM at concentrations of 0.25 mM, 0.75 mM, and 5 mM, respectively. Three oxygen concentrations of 0%, 10%, and 21% O_2_ under each nitrate concentration were realized by replacing the headspace of the cylinder with helium, helium containing 10% (vol/vol) O_2_ (every 3 hours), and air, respectively. For the treatments with a 21% O_2_ concentration, Parafilm with air holes was used to seal the cylinders to maintain aerobic environments, while those for the 0% and 10% O_2_ treatments were sealed using lids with gaskets. All the treatments were repeated three times, and the treatment mixtures were incubated at 30°C with an oscillation rate of 180 rpm.

### Determination of cell growth and nitrate-reducing capacity.

For each treatment, 9 strains were simultaneously inoculated into the corresponding cylinders containing treated FCM, 21 cylinders for each strain. The sampling time points were 0, 6, 12, 18, 24, 30, and 36 h during the incubation, and 3 cylinders were randomly taken for destructive sampling at each sampling point. The sampling procedures were as follows: after being well mixed, 1 mL of the bacterial suspension was collected for monitoring cell density by detecting OD_600_; the rest was centrifuged at 4,000 × *g* for 10 min to separate the supernatant and cell pellet. Then, the supernatant was used for the determination of the sum of NO_3_^−^ and NO_2_^−^ and of NO_2_^−^ concentration by an automatic flow injection analyzer (FIAstar 5000; Foss, Sweden), and the cell pellet was used to measure the cell dry weight after drying for 36 h on a freeze dryer (Neocoole; Yamato). The NO_3_^−^ concentration was gained by subtracting the NO_2_^−^ from the sum of NO_3_^−^ and NO_2_^−^. The biological nitrogen (bio-N) content was detected by using the Kjeldahl method (48) when the strains grew for 12 h under aerobic conditions. Then, the proportion of bio-N in dry cell weight was calculated. The changing amounts of bio-N and nitrate consumption between 0 and 12 h were used to estimate the activities of dissimilatory nitrate reduction and their contributions to nitrate consumption. The amount of nitrate reduced via the dissimilatory process was calculated by subtracting the bio-N from the total nitrate consumption. The contribution of dissimilatory nitrate reduction to nitrate consumption was evaluated by subtracting the proportion of bio-N by cell growth within 12 h. The average growth, nitrate consumption, DNR, and contribution of DNR of three strains of each type were used to characterize the variation pattern between types.

### Statistical analysis.

One-way analysis of variance (ANOVA) with Duncan’s test was performed by SAS (version 9.4) and SPSS software (version 18.0; Chicago, IL, USA) to evaluate the significant difference (*P* < 0.05) of cell growth, nitrate consumption, dissimilatory nitrate-reducing activity, and their contribution. Before the above analysis, the normal distribution of data was checked using a Shapiro-Wilks test. If there is no homogeneity of variance in the data, a nonparametric test is used. All figures were completed by ggplot package in R (version 4.0.5).

### Data availability.

The 16S rRNA gene accession numbers of selected strains are as follows: MW090175 (for P7), MW090176 (for P8), MW090184 (for P16), MW090187 (for P19), MW090194 (for P26), MW090196 (for P28), MW090190 (for P22), MW090197 (for P29), and MW090200 (for P32). The *narG* gene accession numbers are MZ954860 to MZ954865. The *napA* gene accession numbers are MZ954866 to MZ954871.
