# Supplementary material for: The Contribution of Nitrate Dissimilation to Nitrate Consumption in narG- and napA-Containing Nitrate Reducers with Various Oxygen and Nitrate Supplies
Source: Microbiol Spectr. 2022 Dec 1;10(6):e00695-22. doi: 10.1128/spectrum.00695-22 (PMC9769761; doi:10.1128/spectrum.00695-22)
Supplement: Supplemental file 1 — Fig. S1 and S2 and Tables S1 and S2. Download spectrum.00695-22-s0001.pdf, PDF file, 0.3 MB [file spectrum.00695-22-s0001.pdf]

Figure S1. The biological nitrogen of three types of nitrate-reducers at 12 h under varied nitrate and oxygen concentrations.

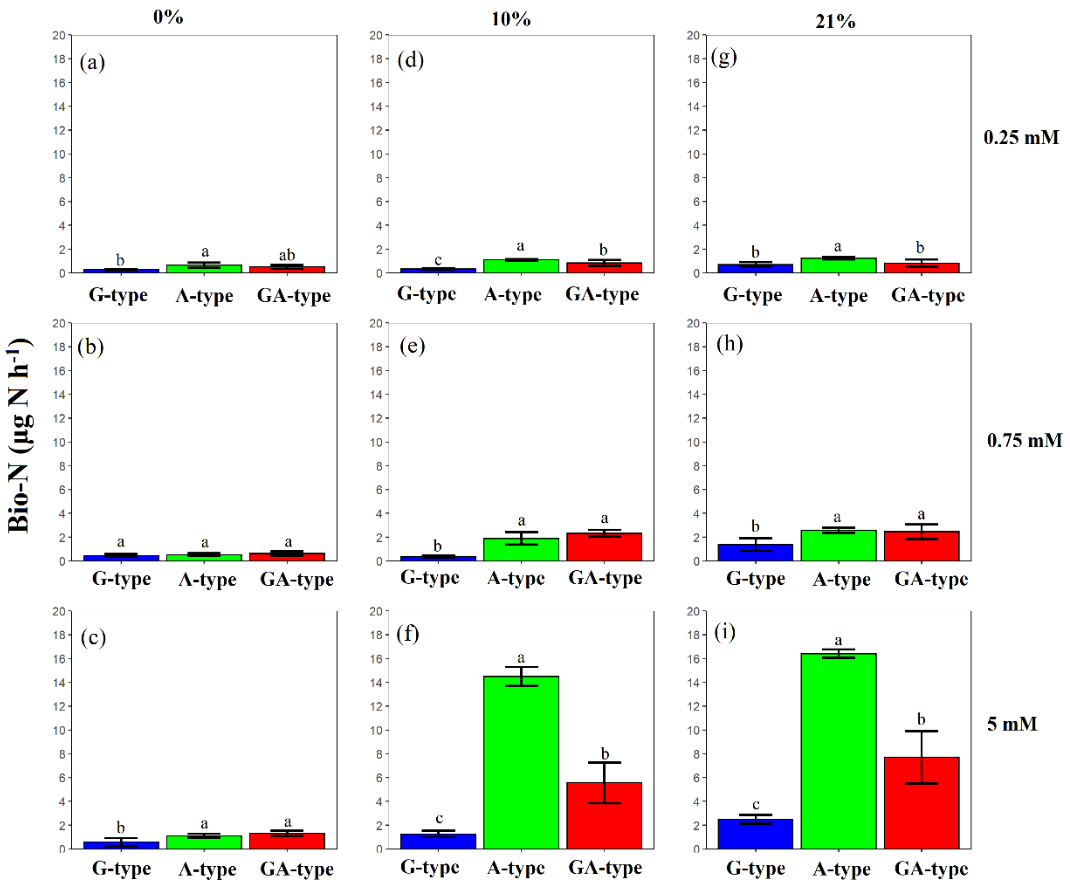

Strains were cultured under anaerobic condition (0% O_2) with 0.25 mM KNO_3 (a), 0.75 mM KNO_3 (b) and 5 mM KNO_3 (c); under facultative condition (10% O_2) with 0.25 mM KNO_3 (d), 0.75 mM KNO_3 (e) and 5 mM KNO_3 (f); under aerobic condition (21% O_2) with 0.25 mM KNO_3 (g), 0.75 mM KNO_3 (h) and 5 mM KNO_3 (i). Standard errors for each type of nitrate reducers (n=9, 3 strains×3 replicates) are given.

Figure S2. Nitrate dynamic of three types of nitrate reducers under varied oxygen and nitrate concentrations.

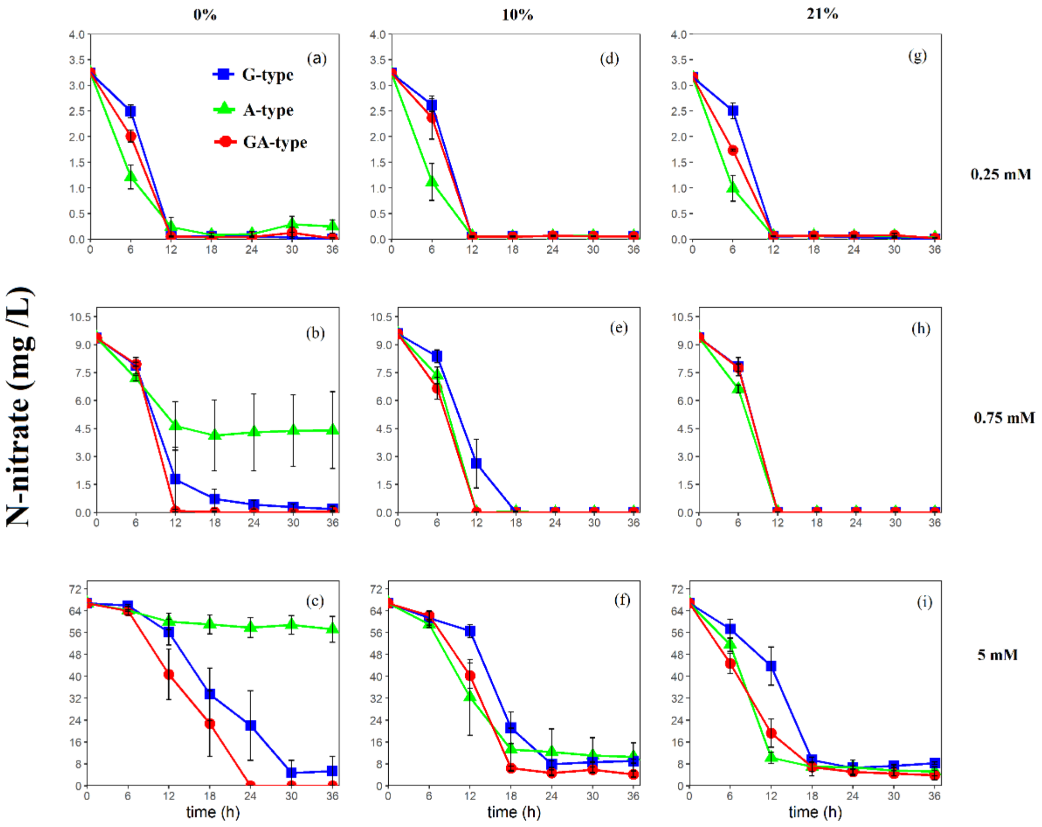

Strains were cultured under anaerobic condition (0% O_2) with 0.25 mM KNO_3 (a), 0.75 mM KNO_3 (b) and 5 mM KNO_3 (c); under facultative condition (10% O_2) with 0.25 mM KNO_3 (d), 0.75 mM KNO_3 (e) and 5 mM KNO_3 (f); under aerobic condition (21% O_2) with 0.25 mM KNO_3 (g), 0.75 mM KNO_3 (h) and 5 mM KNO_3 (i). Standard errors for each type of nitrate reducers (n=9, 3 strains×3 replicates) are given.

Table S1. The dissimilatory nitrate-reducing activities of nine strains of nitrate-reducers under varied nitrate and oxygen concentrations.

Nitrate (mM)		0.25			0.75			5		
Oxygen (v/v)		0%	10%	21%	0%	10%	21%	0%	10%	21%
	P7	9.7ab	13.89a	3.17b	26.06a	23.08a	7.18b	28.76a	27.88a	20.01b
G-type	P8	33.7a	33.10a	21.42b	40.14c	85.94a	60.59b	41.49c	89.98a	58.89b
	P16	17.60a	16.13b	3.36c	49.72a	26.15b	6.29c	85.29a	14.75b	10.65b
	P19	5.64a	1.62b	0.29c	7.83a	3.54c	4.77b	4.35a	1.38b	3.89a
A-type	P26	3.23a	1.21b	0.60c	25.24a	21.29b	2.17c	26.36a	2.96c	4.48b
	P28	17.51a	1.78b	1.48b	19.06a	3.64b	2.74b	10.26a	3.18b	3.77b
	P22	6.46a	1.66b	0.88c	21.13a	2.79b	1.76c	52.68a	9.19b	10.39b
GA-type	P29	39.19a	16.84b	31.77a	118.65a	9.15c	16.48b	157.23a	28.54c	59.09b
	P32	3.87a	1.24b	1.30b	23.69a	3.66b	1.93c	20.87a	4.17b	5.04b

Unit, μg N mg^{-1} dry cell h^{-1}.

Different letters indicate the significant difference (p <0.05) of dissimilatory nitrate reducing activity between different oxygen under the same nitrate concentration condition. The data between 0-12 h were used to estimate the activities of dissimilatory nitrate reduction.

Table S2. The denitrification genes in selected strains.

Isolates	Type	Denitrification genes
P7		*narG*, *nirS*, *norB*, *nosZ*
P8	G	*narG*
P16		*narG*, *nirS*, *norB*, *nosZ*
P19		*napA*
P26	A	*napA*
P28		*napA*
P22		n*arG*, *napA*
P29	GA	*narG*, *napA*, *nirS*, *norB*, *nosZ*
P32		*narG*, *napA*, *nirS*, *norB*, *nosZ*
